# Environmental enrichment improves hippocampus-dependent spatial learning in female C57BL/6 mice in novel IntelliCage sweet reward-based behavioral tests

**DOI:** 10.3389/fnbeh.2023.1256744

**Published:** 2023-09-18

**Authors:** Giulia Bramati, Pia Stauffer, Martina Nigri, David P. Wolfer, Irmgard Amrein

**Affiliations:** ^1^Division Functional Neuroanatomy, Institute of Anatomy, University Zurich, Zürich, Switzerland; ^2^Department of Health Sciences and Technology, ETH, Zürich, Switzerland

**Keywords:** IntelliCage, reward, motivation, environmental enrichment, hippocampus, plasticity, working memory, animal welfare

## Abstract

The IntelliCage is an automated home-cage system that allows researchers to investigate the spontaneous behavior and learning abilities of group-housed mice. The IntelliCage enables us to increase the standardization and reproducibility of behavioral outcomes by the omission of experimenter–mouse interactions. Although the IntelliCage provides a less stressful environment for animals, standard IntelliCage protocols use controlled water access as the motivational driver for learning. To overcome possible water restrictions in slow learners, we developed a series of novel protocols based on appetitive learning, in which mice had permanent access to plain water but were additionally rewarded with sweetened water upon solving the task. C57BL/6NCrl female mice were used to assess the efficacy of these sweet reward-based protocols in a series of learning tasks. Compared to control mice tested with standard protocols, mice motivated with a sweet reward did equal to or better in operant performance and place learning tasks. Learning of temporal rules was slower than that in controls. When faced with a combined temporal x spatial working memory task, sweet-rewarded mice learned little and chose plain water. In a second set of experiments, the impact of environmental enrichment on appetitive learning was tested. Mice kept under enriched environment (EE) or standard housing (SH) conditions prior to the IntelliCage experiments performed similarly in the sweet-rewarded place learning task. EE mice performed better in the hippocampus-dependent spatial working memory task. The improved performance of EE mice in the hippocampus-dependent spatial working memory task might be explained by the observed larger volume of their mossy fibers. Our results confirm that environmental enrichment increases complex spatial learning abilities and leads to long-lasting morphological changes in the hippocampus. Furthermore, simple standard IntelliCage protocols could easily be adapted to sweet rewards, which improve animal welfare by removing the possibility of water restriction. However, complex behavioral tasks motivated by sweet reward-based learning need further adjustments to reach the same efficacy as standard protocols.

## 1. Introduction

Under normal and diseased conditions, behavioral phenotyping allows for the objective and quantitative investigation of complex cognitive processes such as spatial learning and memory, emotionality, and exploratory drive. Classical behavioral tests to investigate such processes were well-established, such as the Morris Water Maze task (Morris, 1981; Vorhees and Williams, 2006) or the open field and elevated mazes (Walsh and Cummins, 1976; Pellow et al., 1985; Shepherd et al., 1994; Stanford, 2007). Despite their effectiveness, these and other classical tests have some limitations. First, the necessary human intervention in classical behavioral testing was a source of stress for the animals, and this affected both animal welfare and the quality of the collected data (Balcombe et al., 2004; Deacon, 2006; Spruijt et al., 2014; d'Isa and Gerlai, 2023). Even simple handling increased the corticosterone levels in rats (Armario et al., 1986). Second, different laboratory environments may lead to differences in behavioral outcomes, even if experimental and/or environmental conditions were strictly standardized (Crabbe et al., 1999; Jaric et al., 2022). To overcome these limitations, new automated phenotyping systems based on video, infrared, radiofrequency identification (RFID) or sensor plates have been developed and used for measurements in home cages (see Voikar and Gaburro, 2020 for a comprehensive overview). These home-cage systems limit the animal–human interaction and concomitantly provide a standardized environment, thereby increasing reliability and eventual reproducibility in future experiments (Krackow et al., 2010; Endo et al., 2011; Spruijt et al., 2014; Kiryk et al., 2020; Grieco et al., 2021). While most of these home-cage systems are designed to test single animals, one exception is the IntelliCage, where up to 16 mice can be tested together (Galsworthy et al., 2005). The IntelliCage (NewBehavior AG and TSE-systems) is an RFID transponder-based, fully automated, and programmable apparatus to study cognitive abilities in group-housed mice over long periods of time (Masuda et al., 2018). Basically, the IntelliCage is a large home cage with four computer-controlled operant chambers fitted into the cage corners. A chamber can be visited by one mouse at a time, while the presence and identity of the animal are registered. Inside the chamber, two water bottles are hidden behind doors. The mouse can access water as a reward by nose poking the door. Whether or not a given door opens after a nose poke depends on the specific task. Once experimental mice are placed in the IntelliCage, they remain in this IntelliCage testing environment with their social group, and all experimental procedures, either on a group or individual level, are managed remotely. Animal activity, measured by visits to chambers, and performance within the chamber were monitored for each animal 24 h a day. This is in stark contrast to classical behavioral tests, where the behavioral responses of animals are usually assessed in novel environments over short periods of time. Many studies pointed out the relevance of the higher sensitivity of IntelliCage testing in detecting exploratory behaviors, circadian rhythm, and learning abilities ranging from simple place learning to complex delay-discounting tasks in wild-type and mutant mice (see Kiryk et al., 2020; Iman et al., 2021 for reviews). Even though the benefits of the IntelliCage test environment are obvious (minimal human intervention, social housing, and a stimulating yet familiar environment), animals with severe learning impairments might become water-restricted, requiring constant monitoring and, if necessary, their removal from the experiment. In line with the three Rs principles (*Reduction, Refinement*, and *Replacement*), we, therefore, sought to overcome the potential consequences of water restriction by creating and testing learning protocols based on a sweetened reward, exploiting the known saccharin preference of C57BL/6 mice (Bachmanov et al., 2001). In these sweet reward-based learning protocols, water was always accessible, while sweet rewards could only be collected if animals made a correct choice. In the first set of experiments, we tested the efficacy and power of the sweet reward-based learning protocols compared to standard protocols, where access to water depended on solving the task correctly. We hypothesized that a sweet reward would be sufficient for several IntelliCage learning tasks but that high cognitive challenges may offset the reward and decrease learning as animals turn to free-access plain water. If so, we wanted to define this turning point and use this information to design modified sweet reward-based protocols.

In the second set of experiments, we explored the effect of environmental enrichment in early adulthood on performance in the sweet reward-based learning paradigms in the IntelliCage. Positive effects of environmental enrichment on animal welfare were well-documented (Bayne, 2018), among other beneficial effects ranging from improvements in spatial learning and memory tests (Frick et al., 2003; Kulesskaya et al., 2011; Hendershott et al., 2016), decreased reward-seeking behaviors (van der Harst et al., 2003; Wood et al., 2006), and neuronal modifications in the hippocampus (Duffy et al., 2001; Hirase and Shinohara, 2014). In this study, female C57BL/6NCrl mice were housed either under standard housing (SH) or enriched environment (EE) conditions and tested afterward on sweet reward-based learning tasks in the IntelliCage.

The sweet-rewarded learning protocols included tests for operant performance, temporal learning, impulsivity, place preference learning, spatial sequence learning (chaining), and reversal learning. After behavioral testing, alterations in brain morphology due to housing conditions were examined by volumetric analysis of the hippocampal fields.

## 2. Materials and methods

### 2.1. Animals

Eight-week-old female C57BL/6NCrl mice (*N* = 56) were obtained from Charles River Laboratories (Sulzfeld, Germany). Mice were group-housed under an inverted light–dark cycle (light on from 20:00 p.m. to 08:00 a.m.). After 1 week of adaptation, mice were injected with a radiofrequency identification (RFID) transponder (Planet ID^®^ GmbH, Germany) under isoflurane inhalation anesthesia (5% isoflurane, 0.7 l/min oxygen). At the age of 10 weeks, mice were randomly assigned to the experimental groups as follows: the Reward-Control experiment (*N* = 24) and the Reward-Housing experiment (*N* = 32). Mice remained in an inverted light–dark cycle for the entire experimental phase. All experimental procedures (introduction into experimental setups, changes in experimental setups, or remote changes in IntelliCage protocols) were performed at ~09:00 a.m., which is 1 h into the animal's dark phase. All animal experiments were conducted under permit No. ZH041/18,29918 of the Canton Zurich Veterinary Office.

### 2.2. Reward-Control experiment

Mice were randomly assigned to the control (*N* = 12) or sweet reward (*N* = 12) group. Each group was tested separately in an IntelliCage equipped with four red shelters, food *ad libitum*, and the operant chambers providing access to water bottles. An extension cage (T3) was permanently connected to each IntelliCage, so mice could be confined either to the IntelliCage or the extension cage while cleaning the other cage. All water bottles in the IntelliCage of the control group contained plain water. In the sweet reward group, each corner of the IntelliCage contained one bottle of plain water and one bottle of sweetened water (saccharin solution: 0.5% saccharin Sigma Aldrich in water). In the sweet reward group, the bottles containing plain water were accessible at any visit in every corner for the entire duration of the experiment, while the bottles with sweetened water were accessible only after a correct response.

Mice were given 3 days of habituation in the IntelliCage (free adaptation, FA), where all doors were open and all bottles were accessible without limitations. In the nose poke adaptation (NPA) phase for operant performance, doors in front of the bottles were closed by default and could be opened for 3 s by a nose poke to the door. In the control group, mice had to perform a nose poke at any door to get access to water. In the sweet reward group, the doors hiding water opened at the beginning of a visit for 3 s without a nose poke, while the doors hiding sweetened water only opened when a nose poke was performed.

In the drinking session adaptation (DSA) phase for time learning, mice could only receive a reward during 4 x 1 h sessions, which were evenly distributed over 24 h. For the control group, water was only accessible during those sessions. Outside the drinking sessions, all doors remained closed. For the sweet reward group, doors hiding saccharin opened after a nose poke during the drinking sessions, while the water doors opened at any visit without a nose poke, regardless of the time of the day.

In the chaining acquisition (CA) task for time x spatial working memory, mice could receive a reward in the corner adjacent to the most recently visited corner in which at least one nose poke had been made. Half of the mice in each IntelliCage had to rotate in a clockwise direction, the other half were assigned to an anti-clockwise direction. As in the task before, the reward (water for the control group, saccharin for the sweet reward group) could only be received upon nose poke in the correct corner during the drinking session. For the sweet reward group, plain water remained available at any time in any corner.

Finally, a recovery phase (with conditions equal to the NPA phase) was followed by a simple place preference (PP) task for place learning, in which each animal could receive water (control group) or saccharin (sweet reward group) in one out of four corners only. Corner assignments were balanced within groups. Again, for the sweet reward group, plain water was accessible in all four corners.

### 2.3. Reward-Housing experiment

**Figure 1:**
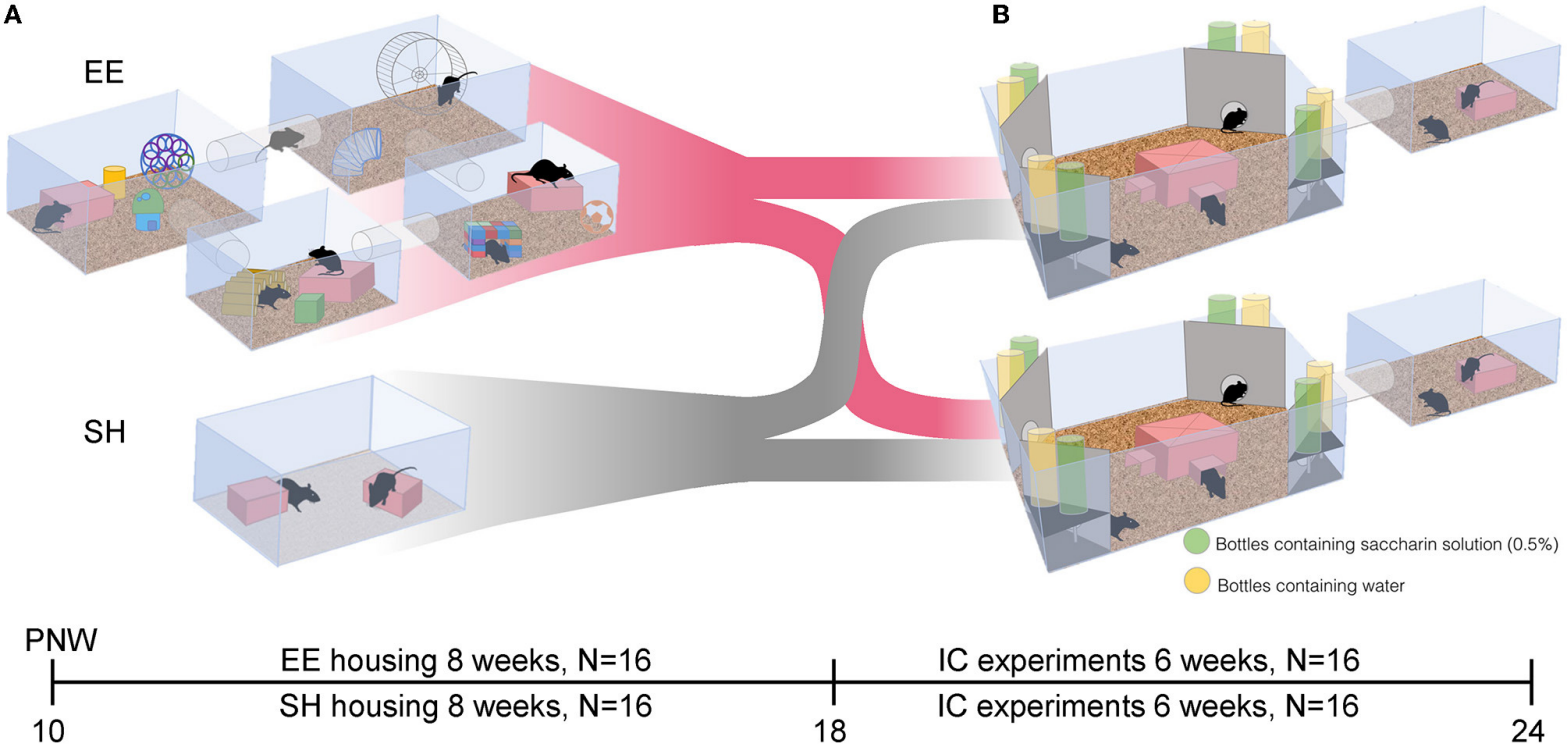
Experimental setup of the Reward-Housing experiment. **(A)** At post-natal week (PNW) 10, female C57BL/6NCrl mice were assigned either to the enriched environment (EE) or the standard housing (SH) condition. In the EE condition, mice were stimulated by a running wheel and the addition and relocation of objects in a system of four tube-connected cages over 8 weeks. At PNW 18, mice from the EE and SH conditions were randomized and transferred into IntelliCage for behavioral testing. **(B)** Behavioral testing was conducted in an IntelliCage (IC, large cage). Corners of the IntelliCage hold experimental chambers where mice had access to free water (yellow bottles) or a sweetened reward (green bottles, 0.5% saccharin in water). Food *ad libitum* was provided in the IntelliCage only, while bedding and mouse shelters were provided in the IntelliCage and the extension cage (tube-connected smaller cage).

At the age of 10 weeks, mice were randomly assigned into environmentally enriched (EE, *N* = 16) or standard-housed (SH, *N* = 16) groups (Figure 1). The setup for the EE mice (eight animals per group) consisted of four tube-connected cages (two T2 and two T3), all provided with water, food, mouse shelters, and nesting material (Figure 1A). A large running wheel was fit into one of the T3 cages. To simulate a dynamically changing environment, eight objects of different shapes, colors, and materials were added once a week to the cages, and one object was relocated. SH mice were housed for 8 weeks in groups of eight animals in normal cages (T3) with water and food pellets *ad libitum*. Cages contained two polycarbonate mouse shelters (ZOONLAB^®^ GmbH) and nesting material (Figure 1A).

After 8 weeks of housing either under EE or SH conditions, mice were again randomized into two groups of 16 animals each (Figure 1B) and tested in two IntelliCage (Figure 1B). In the IntelliCage, test setup and environmental conditions were the same for both groups. For both SH and EE mice, sweet reward protocols were used, that is, free access to water at any time and sweetened water rewards only if mice performed correctly.

The IntelliCage experiments started with the free adaptation (FA) and nose poke adaptation (NPA) phases, followed by the reaction time task (RTT, Kobayashi et al., 2013; Jörimann et al., 2023) to assess impulsivity. After the first nose poke initiated the trial, mice had to withhold the second nose poke for a random delay (between 0.5 and 2.5 s) before a sweet reward was given. Premature nose pokes on the sweet reward door during the delay were punished by trial abortion, and the mouse had to leave the chamber before having the possibility to start a new trial. Prior to this test, two pre-training phases (T0 and T1) were run in which premature nose pokes did not have consequences. The RTT task was followed by an NPA recovery phase. Mice were then trained for the place preference (PP) task for place learning, followed by the spatial working memory chaining acquisition (CA) and chaining reversal (CR) task. In CR, mice had to visit corners in the opposite direction than during CA. The chaining tasks in the Reward-Housing experiment did not include a time component as in the Reward-Control experiment.

### 2.4. Histology

Animals of the Reward-Housing experiment were deeply anesthetized (pentobarbital 50 mg/kg body weight) and perfused transcardiacally with ice-cold phosphate-buffered saline (PBS), followed by sulfide solution and, lastly, with 4% paraformaldehyde (PFA) with 15% picric acid. Brains were removed and post-fixed in PFA + picric acid at 4°C overnight. Left hemispheres were embedded with a 2-hydroxyethylmethacrylate (HEMA)-based polymerizate (Technovit 7100, Kulzer GmbH, Wehrheim, Germany) according to the manufacturer's instructions. Embedded tissue was cut into coronal sections of 20 μm thickness with a rotational microtome (Microm HM325). Every 10th section was collected in a 24-well plate filled with distilled water, mounted in the correct anatomical order on slides, and dried.

For Timm staining, slides were incubated into a developer solution containing gummi arabicum (1:1 in distilled H_2_O), citrate buffer (citric acid and tri-sodium citrate), hydroquinone solution, and AgNO_3_ 34% at 37°C for 40 min. Slides were rinsed in tap water and incubated for 1 min in 1% sodium thiosulfate. After two more washes in distilled water, sections were counterstained by Giemsa solution diluted 1:5 in KH_2_PO_4_ for 15 min at room temperature, dehydrated, and embedded.

### 2.5. Stereological volume analysis of the hippocampus

The volumes of the hippocampal regions were estimated with a design-based stereological method, the Cavalieri estimator (Slomianka, 2021) on the Timm and Giemsa stained sections. Every 10th section containing the hippocampal formation from its rostral to the caudal extent was analyzed using a Zeiss Axio Imager.M2 microscope (magnification 2.5x and 10x) with the Stereo Investigator software 10 (MBF Bioscience, Williston, Vermont USA). Prior to analysis, animal identity was coded. For all regions, a point grid of 100 μm on x- and y-axes was generated and overlaid on each section containing the hippocampus. Seven hippocampal regions were analyzed: granule cell layer of the dentate gyrus (GC); the molecular layer of the dentate gyrus (MOL-DG); hilus of the dentate gyrus (HIL); CA1 including stratum pyramidale, stratum radiatum, oriens, and lacunosum-moleculare; CA3 including stratum pyramidale, stratum radiatum, oriens, and lacunosum-moleculare; subiculum (SUB) and suprapyramidal and infrapyramidal mossy fibers (SI-MF). On average, 15.3 sections (SD = 1.4) in each animal were analyzed.

### 2.6. Statistical analysis

Behavioral data of the Reward-Control and Reward-Housing experiments were exported with the IntelliCage Analyzer software and processed in R (version 4.2.0) for statistical and graphical analyses. Packages used were dplyr, reshape2, lme4, nlme, emmeans, and ggplot2. For statistical analysis, behavioral parameters of each experimental phase were calculated in three time periods: performance on the 1st day, the last day, and aggregated days in between. Repeated ANOVA was used to analyze the main effects of groups and days in each learning phase, including interactions. If the main effects were significant, Tukey's *post-hoc* testing was applied. One-way ANOVA was used to test for group differences in hippocampal volumetric data, followed by Benjamini–Hochberg correction for multiple comparisons (Benjamini and Hochberg, 1995) to adjust *p*-values across all hippocampal regions. The correlation between hippocampal volumetric data and the behavioral parameter was tested with Pearson's correlation. The Shapiro–Wilk normality test was run on behavioral and hippocampal data, and the Box-Cox transformation was applied if necessary. In graphs, untransformed data with mean, SEM, and individual data points in the background are shown.

In the Reward-Housing experiment, two EE mice had to be excluded from the experiment due to elephant teeth (overgrowth of incisors due to the misalignment of mandibular and maxillary teeth) and low drinking (below 100 licks per day) in IntelliCage, respectively. In addition, behavioral data from the 2nd day of RTT (day 16) were excluded from the analysis due to technical problems. Two exclusions of selected data are mentioned in the result section; otherwise, no data were excluded.

## 3. Results

### 3.1. Reward-Control experiment

**Figure 2:**
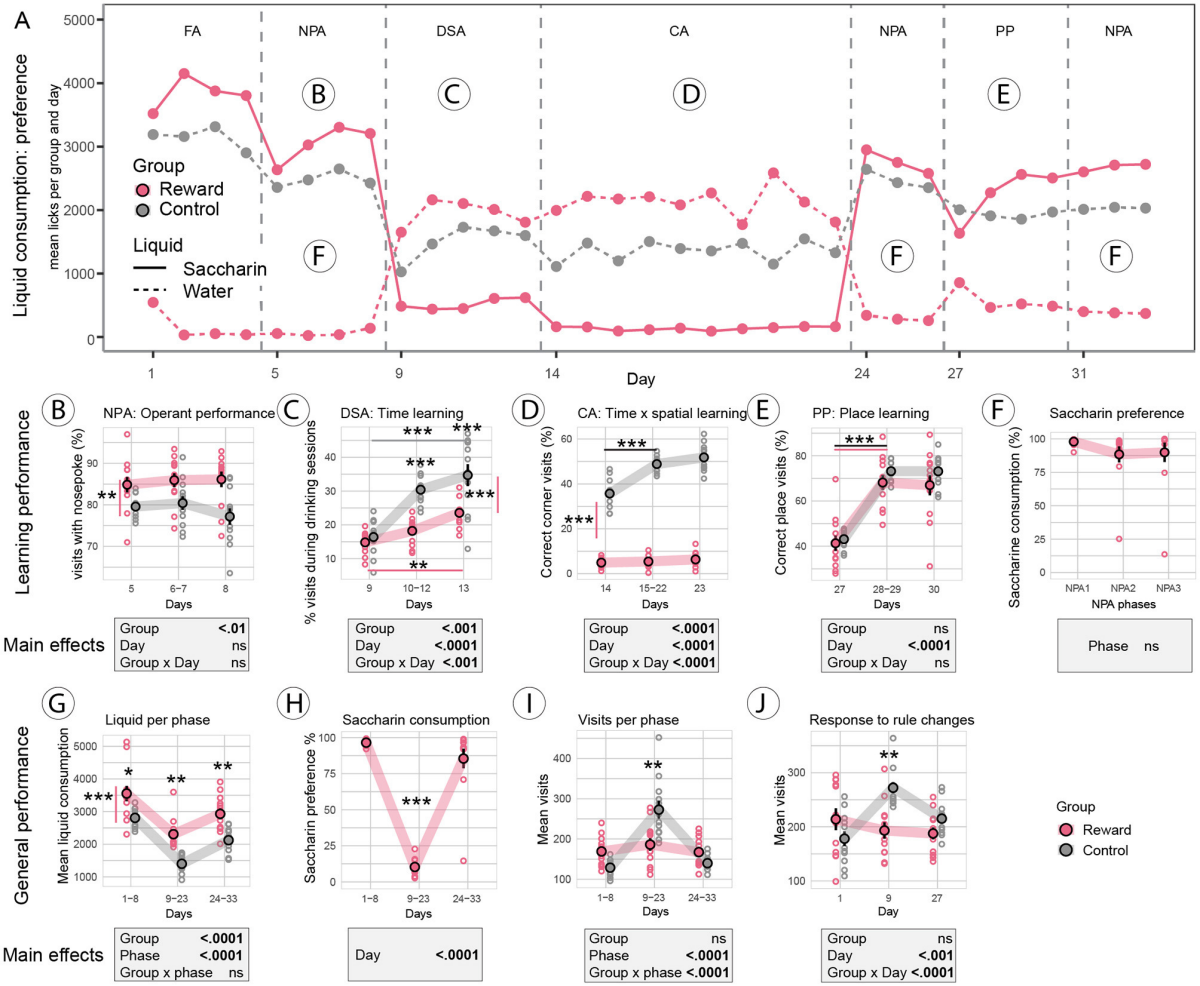
Reward-Control experiment. **(A)** Liquid consumption over the entire experimental phase visualizes tasks where the sweet reward motivated mice engaged in the tasks by drinking saccharin (solid red line high), in comparison to tasks where sweet reward consumption dropped dramatically below free water consumption (dashed red line high). Mice in the control group could only access plain water (dashed gray line). **(B)** Operant performance in the nose poke adaptation (NPA) task was improved in the sweet reward group (*p* < 0.01) compared to controls without a drop in performance over days. **(C)** In the drinking session adaptation (DSA) task, access to water (controls) or saccharin (sweet reward group) was restricted to 4x1 h a day. Mice in the sweet reward group made less visits during drinking sessions (*p* < 0.001) but improved over days (*p* < 0.001). **(D)** In the chaining acquisition (CA) task, rewards were only available during drinking sessions, and rewarded corners rotated after every visit. Mice in the sweet reward group made fewer correct visits (*p* < 0.0001) and could also not improve over days, while control animals showed rapid learning. **(E)** In the place preference (PP) task, rewards were available in only one corner and corner assignments were randomized between mice. Sweet reward and control groups performed equally well; learning was significantly improved after the 1st day of testing (*p* < 0.0001). **(F)** Saccharin consumption in the sweet reward group remained stable in the three NPA phases. Note that two mice (outliers in NPA2 and NPA3) increasingly preferred free water over saccharin. Under standard protocols, these two mice would most likely have to be excluded from the experiment. **(G)** Total consumption of liquid (saccharin plus water) increased in the sweet reward group throughout the experiment (*p* < 0.0001). **(H)** Saccharin consumption dropped dramatically during the DSA and CA tests in the sweet-rewarded group. **(I)** Mean number of visits was higher in controls than in sweet-rewarded mice during the challenging DSA and CA tasks (days 9–23, *p* = 0.001). **(J)** Novelty response, assessed as the number of visits during days of rule change, was higher in controls when a challenging task was introduced (day 9, start of DSA task, *p* = 0.006). Mice in the sweet reward group did not increase activity due to rule changes. ^***^: *p*-value < 0.001; ^**^: *p*-value < 0.01; ^*^: *p*-value < 0.05.

Visualization of the consumption of saccharin vs. water over the entire experimental phase provides a general survey of animal performance and task complexity (Figure 2). Overall, appetitively motivated mice consumed more liquid (saccharin plus water) than controls (water only) over the entire experimental phase [*F*_group (1, 22)_ = 26.9, *p* < 0.0001, Figure 2G]. Mice in the sweet reward group showed a strong preference for saccharin consumption in phases when tasks were simple (days 1–8, days 24–33, Figures 2A, H). However, saccharin consumption dropped dramatically in the phase of challenging tasks [*F*_phase (2, 22)_ = 157.2, *p* < 0.0001, Figure 2H]. There was no evidence for a difference between the control and sweet reward groups for general activity, assessed as the mean number of corner visits per day [*F*_group (1, 22)_ = 0.2, *p* = 0.7]; however, there was evidence for a phase effect [*F*_phase (2, 44)_ = 44.8, *p* < 0.0001]; *post-hoc* analysis revealed an increase in activity (*p* = 0.001) in the phase of challenging tasks (days 9–23) in control mice (Figure 2I). To check for novelty responses, we analyzed visit activity as a response to environmental or rule changes, that is, during the 1st day of the IntelliCage experiment, the 1st day of the DSA protocol, and the 1st day of the PP protocol. A significant group difference could only be established for the 1st day of DSA (*p* = 0.006, Figure 2J) where control mice increased their visits; otherwise, groups responded similarly to novelty.

Simple learning was analyzed during NPA and PP. The sweet rewards improved operant performance in the NPA phase (Figure 2B) in the sweet reward group compared to controls [*F*_group (1, 22)_ = 10.16, *p* < 0.01]. Data suggested that sweet rewards prevented a drop in performance on the last day of NPA in the reward group (Figure 2B). In the simple place preference task (PP, Figure 2E), both the sweet reward and control groups learned to visit the rewarded corner equally well and correct corner visits increased over time [*F*_day (2, 46)_ = 211.8, *p* < 0.0001]. *Post-hoc* comparison for each time point revealed that the performance gain was achieved after the 1st day in both groups (*p* < 0.0001, Figure 2E).

In the complex learning tasks, the sweet reward group preferred free water over saccharin; nevertheless, the analysis of performance revealed some improvements. Time learning in the drinking adaptation phase (DSA, Figure 2C) was analyzed by the percentage of visits during drinking sessions. Effect size was different for groups [*F*_group (1, 22)_ = 19.5, *p* < 0.001], time points [*F*_day (2, 44)_ = 53.9, *p* < 0.0001], and interaction [*F*_groupxday (1, 44)_ = 9.8, *p* < 0.001]. *Post-hoc* analysis suggested that controls performed better than the sweet reward group after the 1st day (*p* < 0.001); however, the sweet reward group improved over time (*p* < 0.001). In the chaining acquisition task (CA, Figure 2D), which is a combined time x spatial working memory task, the sweet reward group performed markedly worse than the control group [*F*_group (1, 22)_ = 855.1, *p* < 0.0001]. *Post-hoc* testing revealed that controls improved after the 1st day (*p* < 0.0001), while in the sweet reward group, there was no evidence of an improvement over time.

In the sweet reward group, consumption of free available water during the three NPA phases was low (on average between 1 and 11%). Importantly, saccharin consumption during the three phases of NPA did not decline significantly over time [*F*_phase (2, 46)_ = 2.05, *p* = 0.14, Figure 2F], suggesting that the attractiveness of the sweetened reward did not change over time. Note that two mice of the sweet reward group lost preference for saccharin over time (outliers in Figure 2F) and increased plain water consumption.

### 3.2. Reward-Housing experiment

**Figure 3:**
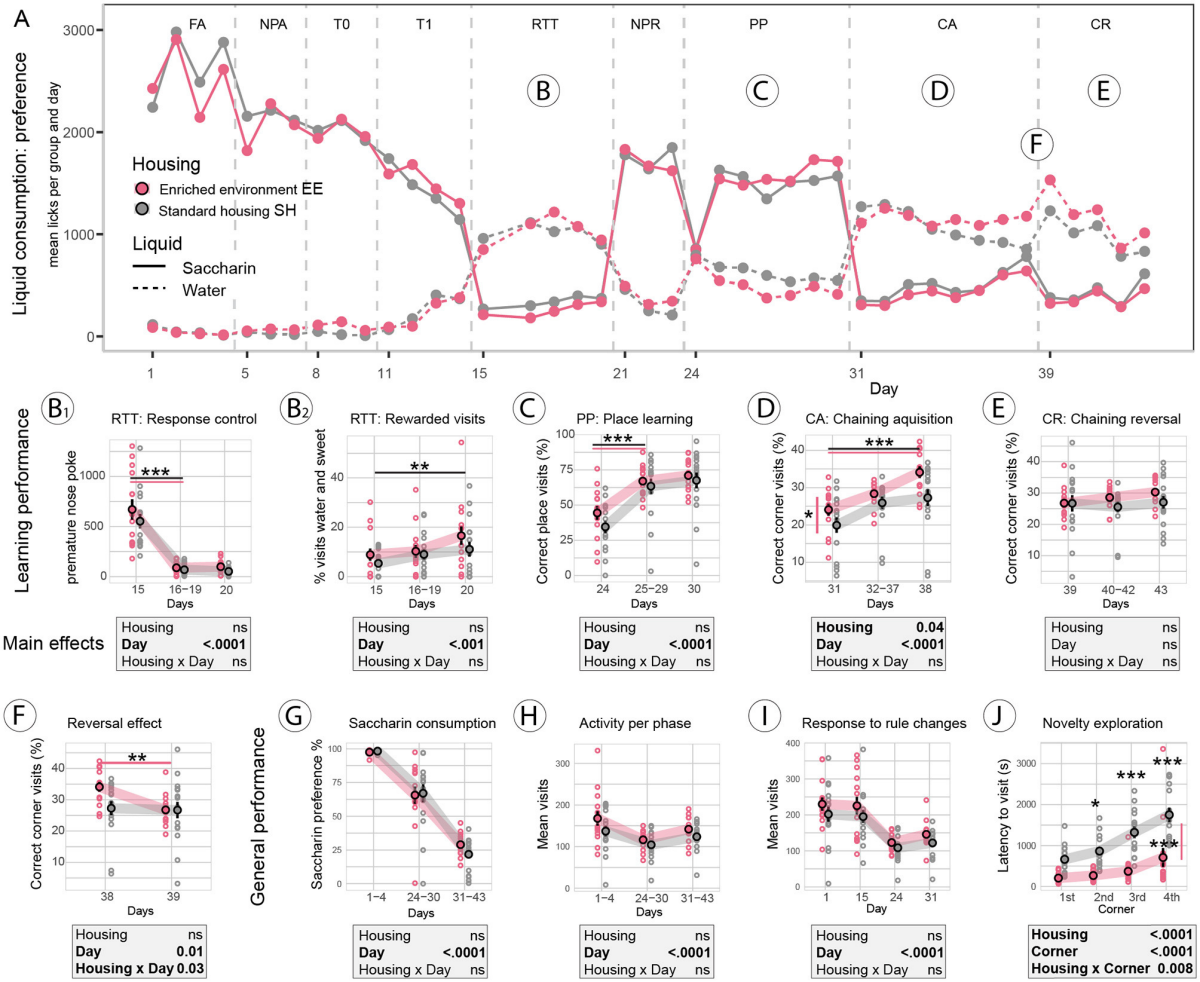
Reward-Housing experiment. **(A)** Overview of saccharin (solid line) vs. water (dashed line) consumption in the enriched environment (EE, red) and standard-housed (SH, gray) groups. Saccharin consumption dropped below water consumption in complex learning tasks. **(B)** In the reaction time task (RTT) assessing impulsivity, **(B2)** mice of both groups learned equally well to withhold premature nose pokes after the 1st day of testing (*p* < 0.0001). **(B2)** Detailed analysis of visits revealed that the percentage of visits in which mice consumed both water and saccharin increased over time (day effect *p* = 0.001), indicating that mice learned a workaround in this task. **(C)** In the place preference (PP) task, no group difference was found; all mice learned to visit the correct corner after the 1st day (*p* < 0.0001). **(D)** Correct corner visits were improved in EE mice (*p* = 0.04) in the chaining acquisition (CA) task; over days, both EE (*p* < 0.0001) and SH (*p* < 0.001) improved. **(E)** In the chaining reversal (CR) task, none of the groups could improve. **(F)** In the analysis of the reversal effect, comparing the last day of CA to the 1st day of CR, we found a group x day interaction (*p* = 0.03), the effect was due to the EE mice performing worse after the rule change (*p* = 0.008), while correct corner visits in SH mice were indifferent to the rule change. **(G)** General assessment of sweet reward consumption revealed that saccharin preference did not differ between groups. **(H)** Visit activity during easy or complex phases did not differ between groups. **(I)** Visit activity in response to the rule change did not differ between groups. **(J)** However, novelty exploration, measured as the latency to visit all four corners of the IntelliCage for the first time at the beginning of the experiments, showed faster exploration in the EE group (*p* < 0.0001). ^***^: *p*-value < 0.001; ^**^: *p*-value < 0.01; ^*^: *p*-value < 0.05.

In this experiment, standard and enriched environment-housed mice had access to saccharin as a sweet reward for correct performance, while plain water was always available. A graphical overview of liquid consumption over the entire experimental period indicates which tasks were difficult for the mice to solve (Figure 3A). The main focus of the analysis was on group differences due to housing conditions, e.g., between the enriched environment (EE) and standard-housed (SH) groups. Over the entire experimental phase, there was no indication of a different preference for saccharin over water between housing groups [*F*_group (1, 28)_ = 0.4, *p* = 0.5], as shown in the selected analysis of FA, PP, and CA/CR phases (Figure 3G). As observed in the Reward-Control experiment before, saccharin consumption dropped dramatically in the challenging RTT and CA/CR phases (Figures 3A, B, G). Overall activity (visits per day) was indistinguishable between groups [*F*_group (1, 28)_ = 1.9, *p* = 0.2], confirmed by the analysis of selected experimental phases (Figure 3H).

Furthermore, there was no evidence for a group difference in novelty responses, analyzed by visit activity as a response to environmental or rule changes, that is the 1st day of Intellicage testing and the 1st days of RTT, PP, and CA (Figure 3I). Novelty exploration, assessed at the start of the experiment as the latency to visit the IntelliCage corners for the first time, revealed faster exploration in the EE group [*F*_group (1, 27)_ = 31.0, *p* < 0.0001], and *post-hoc* analysis indicated that the latency to visit the second, third, and fourth corners was shorter in EE-housed mice (Figure 3J). In this analysis, one SH mouse was an extreme outlier (latency by more than two SD higher than the mean) and was excluded from the analysis.

Analysis of learning was performed for the RTT, PP, and chaining tasks. In the RTT task assessing impulsivity (Figure 3B1), both groups scored equally high in premature poke repetition on the 1st day and learned to withhold repetitive poking on the saccharin door in the following days [*F*_day (2, 56)_ = 76.3, *p* < 0.0001]. Liquid consumption, however, indicated that mice mainly switched to water consumption (see Figure 3A). Saccharin consumption remained low, and most of the sweet rewards (62%) were received after the shortest delay of 0.5 s (data not shown). However, mice also found a workaround in this test. Detailed analysis suggested that mice increasingly consumed both water and saccharin during the same visit [*F*_day (2, 56)_ = 7.4, *p* = 0.001] by initiating the trial with a first (correct) nose poke to the saccharin door, then switching to free water consumption, and, once the delay was over and the sweet reward door opened, also consuming saccharin water (Figure 3B2).

In the PP task (Figure 3C), both groups improved over time [*F*_day (2, 56)_ = 111.0, *p* < 0.0001], without evidence for a group difference. The chaining acquisition (CA) task in this experiment was designed as a spatial working memory task without a time component, that is, correct responses required visiting corners consecutively in a clockwise or anti-clockwise fashion without restriction to specific time windows. Mice improved with correct corner visits over time [*F*_day (2, 56)_ = 27.5, *p* < 0.0001, Figure 3D], and EE animals performed overall better in this task [*F*_group (1, 28)_ = 4.6, *p* = 0.04]. In the chaining reversal (CR) task (Figure 3E), the direction of the rewarded corners for each animal was reversed. The recovery of correct performance for the group or time during the CR task was not significant. To investigate the reversal effect in response to the spatial rule change, performance during the last day of CA and the 1st day of CR was compared (Figure 3F). Correct corner visits declined [*F*_day (1, 28)_ = 6.7, *p* = 0.01], and *post-hoc* comparison indicated that the decline was due to the drop in performance of EE mice (*p* < 0.01), indicating that SH mice did not show a reversal effect as performance remained on chance level (~25% of correct corner visits), both during the last day of CA and the 1st day of CR. After the rule change, the performance of EE mice was on a chance level too.

### 3.3. Larger mossy fibers correlate with the reversal effect in the spatial sequence task

**Figure 4:**
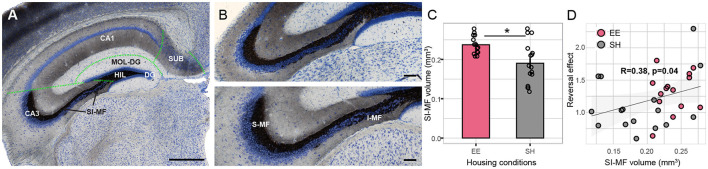
Mossy fiber volume correlates with housing conditions. **(A)** Representative Timm stained, coronal section of the hippocampal formation, counterstained with Giemsa. Volumetric analysis was performed for the dentate gyrus granule (DG) cell layer, dentate gyrus molecular layer (MOL-DG), and hilus (HIL). In the CA3 and CA1 regions, pyramidal layers and associated layers of stratum oriens, radiatum, and lacunosum-moleculare were pooled for the volumetric analysis. Lastly, subiculum (SUB) and supra-infrapyramidal mossy fibers (SI-MFs) were measured. Green dashed lines indicate the boundaries of hippocampal fields. **(B)** Enlarged view of the suprapyramidal (S-MF) and infrapyramidal (I-MF) mossy fibers in the dorsal region of the hippocampus of SH (upper panel) and EE (lower panel) mice. **(C)** Total volume of SI-MF was larger in EE mice compared to SH mice (*p* = 0.036); on average, 15 sections per animal were analyzed, spanning the entire rostral to the caudal axis of the hippocampus. **(D)** A significant positive within-group correlation between SI-MF size and reversal effect after the rule change was found (*p* = 0.04); reversal effect was calculated as the percentage of correct corner visits on the last day of the CA task divided by the same parameter on the 1st day of CR. Reversal effects larger than 1 indicate learning of the previous spatial sequence rule and a drop in performance after the rule change. EE, Enriched environment; SH, standard housing; Scale bar A = 500 mm, B = 100 mm. ^*^: *p*-value < 0.05.

Hippocampal fields (Figure 4A) were analyzed using the Cavalieri method. The precision of the volumetric estimations was tested by calculating the coefficient of error (CE) with a smoothness constant of m = 0 (Gundersen and Jensen, 1987; Slomianka and West, 2005). CE was low and varied between 0.03 and 0.13 (Table 1). The ratio CE over the relative group variance was smaller than 0.5 (range 0.1–0.3), indicating that measurement precision did not limit our ability to detect volumetric changes between groups (Slomianka, 2021). There was no evidence for differences between SH and EE mice in the volume of the dentate gyrus granule cell layer, dentate gyrus molecular layer, hilus, CA1, CA3, and subiculum. However, we found a housing effect in the volume of the terminal field of the mossy fibers (Figures 4B, C and Table 1).

**Table 1:** Cavalieri estimations of hippocampal volumes and group statistics.

	**Estimated volume (mm** ^**3**^ **)**				**Group statistics**			**Estimate precision**
**Hippocampal fields**	**EE mean**	**EE SD**	**SH mean**	**SH SD**	***p*** **-value**	***F***	***p*** **-adjusted (BH)**	**CE (m** **=** **0)**
Dentage gyrus granule cell layer	0.30	0.02	0.29	0.03	0.97	0.00	0.971	0.06
Dentage gyrus molecular layer	1.18	0.13	1.16	0.11	0.70	0.15	0.971	0.03
Hilus	0.18	0.03	0.16	0.03	0.24	1.43	0.969	0.13
CA3	1.64	0.19	1.65	0.14	0.85	0.03	0.971	0.05
CA1	2.64	0.26	2.65	0.25	0.97	0.00	0.971	0.03
Subiculum	1.42	0.14	1.41	0.16	0.89	0.02	0.971	0.04
Mossy fiber supra/infrapyramidal (SI-MF)	0.24	0.02	0.19	0.05	**0.005**	9.56	**0.036**	0.08
Hippocampus total	7.59	0.69	7.52	0.59	0.76	0.09	0.971	-

Unilateral volumetric measurements.

EE, enriched environment-housed group; SH, standard-housed group; SD, standard deviation; BH, Benjamini–Hochberg correction for multiple comparisons; CE, coefficient of error; m, smoothness factor.

Significant group comparisons are indicated in bold.

The suprapyramidal and infrapyramidal mossy fibers (SI-MF) were larger in the EE group [*F*_group(1, 28)_ = 9.6, *p* = 0.005, adjusted for multiple comparisons *p* = 0.036, Table 1]. We tested SI-MF volume against the reversal effect in the CA to the CR task (see Figure 3F). The reversal effect was expressed as the percentage of correct corner visits on the last day of the CA task (day 38) divided by the same parameter on the 1st day of CR (day 39).

Hence, reversal effects larger than 1 indicated learning of the previous rule and a drop in performance after the rule change. Data indicated a significant within-group correlation of SI-MF volume with the reversal effect (*t* = 2.1, df = 28, *p* = 0.04, Figure 4D), suggesting that mice with larger SI-MF showed an increased reversal effect.

## 4. Discussion

Automated home-cage systems provide powerful tools for the reproducible and standardized assessments of spontaneous behavior and cognitive abilities in laboratory rodents (Spruijt et al., 2014; Voikar and Gaburro, 2020; Grieco et al., 2021). Of the currently commercially available systems, the IntelliCage is the only home-cage system for high-throughput screening of the behavioral performance of group-housed mice. We tested the benefits and limitations of sweet reward-based tests in the IntelliCage while avoiding water restriction, thus improving animal welfare in this automated behavioral phenotyping system. The predilection of C57BL/6 mice for saccharin over a wide range of concentrations (0.1–20.5 g/l, Bachmanov et al., 2001) was exploited in the present study as a sweet reward-based driver for learning. Saccharin was preferred over sucrose because of the metabolic effects implied by the prolonged consumption of the latter on body weight and enzymatic activity (Black et al., 1998). Compared to controls, which could gain only water as a reward, mice with sweet rewards were more eager to engage in the tasks, as they showed increased liquid consumption over the entire period of testing, without a significant drop in saccharin preference over time.

### 4.1. Efficiency of sweet reward-based learning in the IntelliCage

The sweet reward-based protocols can easily be applied in tests for explorative behaviors and circadian rhythm, since the behaviors observed during FA and NPA were consistent with those achieved with standard protocols. Furthermore, our findings indicated that, in operant performance, place learning and, to some extent, time learning, sweet reward protocols did not compromise learning efficiency while improving animal welfare. However, the chaining task, combining time learning with spatial working memory learning, posed too much of a challenge for the sweet reward group. These mice switched to plain water consumption and did not engage in learning the task. We used this finding to redesign the chaining protocol for the Reward-Housing study, where the time component was removed from the spatial working memory task. Even though performance at the end of this version of the chaining task was below controls, both SH and EE mice learned the task by significantly improving over time. The reaction time task (RTT, Kobayashi et al., 2013) assesses impulsivity and motor response control. Mice receiving sweet rewards learned to withhold premature nose pokes on the saccharin door. However, data indicated that successful inhibition of nose pokes did not increase sweet reward consumption; rather, mice switched mainly to plain water or found a workaround by switching to water consumption to pass the delay time. As observed in our study, mice are not prone to waiting. A decline in the willingness to wait for a sweetened reward was already apparent in the training phases preceding the RTT test, where the rewarding stimulus lost attractiveness even before premature nose pokes had negative consequences. The RTT task in the IntelliCage is a powerful test to detect impulsivity in mice (Kobayashi et al., 2013; Masuda et al., 2016; van Dijk et al., 2016). However, the test is quite challenging for the animals (Jörimann et al., 2023). Modifications to the current sweet reward-based RTT protocol would be highly desirable. Alterations could be achieved by making either saccharin more attractive and water less appealing or by preventing double takes of water and a sweet reward during the same visit.

### 4.2. Environmental enrichment improves complex spatial learning

Differences in behavior between the EE- and SH-housed mice were observed for the learning complex spatial rules and a reversal effect after the rule change, while both experimental groups displayed the same ability in simple place learning. Improvement in spatial learning and retention after environmental enrichments, usually assessed in the Morris water maze (MWM), is well-documented (Kempermann et al., 1998; Wolfer et al., 2004; Leggio et al., 2005; Bennett et al., 2006; Nithianantharajah and Hannan, 2006; Hüttenrauch et al., 2016, to name but a few). Hippocampal lesion experiments have shown that both MWM and various forms of spatial sequence learning in the IntelliCage are hippocampus-dependent learning tasks (D'Hooge and De Deyn, 2001; Voikar et al., 2018). Spatial learning and memory processes in the hidden platform version of the MWM can be based on different strategies using extramaze cues, proximal cues, or praxis (learning a sequence of movements, Janus, 2004). Spatial sequence learning tasks in the IC, such as the chaining task or the patrolling task (Onishchenko et al., 2007; Albuquerque et al., 2013), might depend less on extramaze cues as the IntelliCage is smaller and relatively enclosed. In addition, mice will be predominantly active during the dark phase when local cues might be more relevant. Moreover, correct performance in spatial sequence tasks in the IntelliCage depends on spatial working memory, as correct corner visits are predictable based on the location of the previous correct visit. Spatial sequence learning tasks in the IntelliCage are more similar to the 8-arm radial maze used to assess spatial working memory (Reinstein et al., 1983). In this study, mice in the EE group performed better than SH mice in the acquisition phase of the spatial working memory-dependent chaining task and showed a stronger reversal effect after the rule change in the chaining reversal phase. In contrast to the chaining task, EE had no effect on simple place preference learning, both groups were equally successful in learning this task. Place recognition, necessary to solve the place preference task in the IntelliCage, is hippocampus-independent, as hippocampal lesion experiments have shown before (Voikar et al., 2018). Stimulation with a dynamically changing environment prior to the IntelliCage experiments had no impact on place recognition abilities. EE conditions might affect higher spatial skills and more complex aspects of spatial memory, leading to the formation of more intricate cognitive maps necessary to learn adaptive spatial rules, as in the chaining task.

### 4.3. Enlarged suprapyramidal and infrapyramidal mossy fibers after environmental enrichment

Providing a stimulating environment that fits species-specific needs improves the wellbeing of laboratory rodents (summarized by Smith and Corrow, 2005; Neville et al., 2023). It has been shown that the benefits of EE for wild-type rodents and animal models of brain disorders are multilevel, encompassing visual, motor, cognitive, and somatosensory systems (for a review, see Nithianantharajah and Hannan, 2006). The expression of genes related to synaptic function and cellular plasticity is altered in the cortex and hippocampus of mice reared under enriched conditions (Rampon et al., 2000a; Hüttenrauch et al., 2016). Morphological changes in the hippocampus after EE include increased synapse density in the CA1 region (Rampon et al., 2000b) and a larger cell size of pyramidal neurons in CA1 with longer dendrites in the CA1 and dentate gyrus (Faherty et al., 2003). EE in C57BL/6 mice over 11 months increases the number of dentate gyrus granule cells and leads to a volumetric increase of the cell layers of the dentate gyrus and CA1 (Hüttenrauch et al., 2016). Furthermore, EE promotes adult neurogenesis of granule cells in laboratory rodents (Kempermann et al., 1997), and axonal growth of the newly born neurons preferentially contributes to the infrapyramidal mossy fiber field, leading to a net increase of infrapyramidal mossy fibers after enriched conditions (Römer et al., 2011). In the present study, EE for 8 weeks prior to the IntelliCage behavioral experiments did not lead to a volumetric change in the cell layer of the dentate gyrus. However, we found a persisting volumetric increase of the suprapyramidal and infrapyramidal terminal fields of the mossy fibers in EE mice, while none of the other hippocampal fields showed volumetric changes in the EE mice compared to SH mice. Our finding of enlarged mossy fiber terminal fields due to EE is supported by evidence both for the suprapyramidal and infrapyramidal regions. EE increases the number, size, and complexity of local terminal arborization complexes of mossy fibers, as well as synapse number and dendritic spine length in the suprapyramidal mossy fiber field (Galimberti et al., 2006; Gogolla et al., 2009). Even though we did not separately assess suprapyramidal vs. intrapyramidal and infrapyramidal mossy fibers, it is intriguing to note that larger intrapyramidal and infrapyramidal mossy fibers have been associated with more efficient navigation strategies in the MWM and radial maze (Crusio et al., 1987; Pleskacheva et al., 2000), as well as increased retention in the MWM (Schöpke et al., 1991), suggesting that larger mossy fibers stabilize ongoing behavior and facilitate the processing and use of complex spatial information (Crusio, 2001). This corresponds well with our observation of better performance in the spatial working memory task and increased reversal effects in EE mice. Moreover, within-group covariance analysis revealed a significant positive association between the reversal effect and suprapyramidal and infrapyramidal mossy fiber sizes.

### 4.4. Explorative behavior and reward-seeking behavior in appetitively motivated learning tasks

Based on previous studies underlining the positive influence of environmental enrichment on explorative behavior by mitigating anxiety-like behaviors (Chapillon et al., 1999; Moreno-Jiménez et al., 2019), we expected to observe increased explorative behavior in EE mice in the IntelliCage. Enrichment did, indeed, lead to shorter latency in exploring the IntelliCage at the beginning of the experiments. However, exploratory behavior in response to rule changes during the following experimental phases was not significantly higher in the EE group, possibly due to an environmental habituation effect. Alternatively, the IntelliCage itself can be considered a form of environmental enrichment (see Figure 1), as it provides both social interactions as well as increased physical activity in a complex environment. The continuous IntelliCage enrichment could have compensated for the previous housing conditions for SH mice. However, the IntelliCage enrichment did not mask the improved spatial working memory performance of mice exposed to the EE condition. Thus, IntelliCage experiments are still a suitable tool to study the effects of previous EE on cognitive performance. Appetitively motivated learning depends on the equal and continuous attractiveness of the reward for both experimental groups. A large body of evidence shows that seeking behavior declines under EE conditions, in particular concerning substances of abuse (Stairs and Bardo, 2009; Olsen, 2011). Our findings in female C57BL/6 mice indicated that preference for sweet rewards was not different between the EE and SH groups, which is in agreement with previous reports of equal sucrose preference in male C57BL/6 mice under EE or social housing conditions, while ethanol preference was reduced in EE mice (Holgate et al., 2017).

In summary, we showed that phenotyping mice in the IntelliCage can be improved further in terms of animal welfare by introducing sweetened water as a reward, while always providing the option to drink plain water, avoiding water deprivation in slow learners. A sweet reward as a motivational driver in the IntelliCage is sufficient to induce robust operant performance and simple place learning. Significant spatial sequence learning and time learning can be achieved with sweet reward-based learning, although the extent is smaller than in standard protocols. Sweet reward-based motivation is not sufficient to induce complex spatial sequence × time learning or successful performance in the impulsivity task. In the present study, only female mice were tested. Previously, female or male mice have been tested using sweet rewards for preference or place learning in the IntelliCage (Kiryk et al., 2020). When both sexes have been investigated, no sex difference in sweet reward preference has been reported (Morello et al., 2020). This is in contrast to conventional saccharin consumption studies, where male C57BL/6 mice showed higher intake compared to female mice (control animals in Di Segni et al., 2019). A formal test of sex-dependent performance in sweet reward-based learning tasks in the IntelliCage is currently missing and could be the subject of future research. Finally, additional studies testing refined IntelliCage protocols might improve the effectiveness of sweet reward-based learning on complex tasks.

## Data availability statement

The raw data supporting the conclusions of this article will be made available by the authors, without undue reservation.

## Ethics statement

The animal study was approved by Canton Zurich Veterinary Office, Switzerland. The study was conducted in accordance with the local legislation and institutional requirements.

## Author contributions

GB: Investigation, Formal analysis, Writing—original draft. PS: Investigation, Writing—original draft. MN: Methodology. DPW: Resources, Formal analysis, Supervision, Writing—review and editing. IA: Conceptualization, Formal analysis, Supervision, Writing—review and editing.
